# Comparing the measurement properties of the EQ-5D-Y-3L and EQ-5D-3L in a general population sample of adults

**DOI:** 10.1007/s10198-025-01839-7

**Published:** 2025-10-17

**Authors:** Anna Nikl, Valentin Brodszky, Ákos Szabó, Fanni Rencz

**Affiliations:** 1 Semmelweis University Doctoral Collage, 26 Üllői út, Budapest, H-1085 Hungary; 2 Department of Health Policy, Corvinus University of Budapest, Budapest, Hungary; 3 Department of Financial Accounting, Corvinus University of Budapest, Budapest, Hungary; 4 EuroQol Research Foundation, Rotterdam, The Netherlands

**Keywords:** EQ-5D-3L, EQ-5D-Y-3L, Self-reported health, Descriptive systems, I10

## Abstract

**Objectives:**

EQ-5D has separate three-level versions for children/adolescents (EQ-5D-Y-3L) and adults (EQ-5D-3L), assessing the same five dimensions of health-related quality of life (HRQoL) using age-appropriate language. Little is known about how differences in wording affect self-reported HRQoL assessments. This study aimed to compare the measurement properties of the EQ-5D-Y-3L and EQ-5D-3L in an adult general population sample.

**Methods:**

A cross-sectional study was conducted in a Hungarian adult general population sample representative by age and gender (*n* = 1,196). Measurement properties, including ceiling, floor, informativity, agreement (Kendall’s tau) and known-groups validity (effect sizes) based on self-perceived health and chronic conditions were compared across instruments.

**Results:**

EQ-5D-Y-3L and EQ-5D-3L yielded 85 and 47 unique health states, respectively. Identical health profiles were reported by 59.0%. Overall ceiling was lower using the EQ-5D-Y-3L (34.8%) than the EQ-5D-3L (46.8%), with the largest dimension-level difference for EQ-5D-Y-3L worried/sad/unhappy (56.8%) vs. EQ-5D-3L anxiety/depression (71.6%). Relative informativity was higher for all EQ-5D-Y-3L dimensions (0.20–0.75) than EQ-5D-3L (0.18–0.66). Agreement was the weakest for worried/sad/unhappy vs. anxiety/depression (0.636) and the strongest for mobility (0.841). Both instruments showed medium to large effect sizes across known-groups based on level sum scores (EQ-5D-Y-3L: 0.820–2.454; EQ-5D-3L: 0.820–2.696) and index values (EQ-5D-Y-3L: 0.754–2.362; EQ-5D-3L: 0.747–2.365), with EQ-5D-Y-3L showing higher discriminatory power in 62–69% of known groups.

**Conclusion:**

Notable differences emerged between EQ-5D-Y-3L and EQ-5D-3L in an adult general population sample, especially in the mental health dimension, suggesting that transitions between these instruments should be treated cautiously. The EQ-5D-Y-3L may offer advantages in detecting variations in mental health, even in adult populations.

**Supplementary Information:**

The online version contains supplementary material available at 10.1007/s10198-025-01839-7.

## Introduction

Health-related quality of life (HRQoL) has become a central focus in evaluating the impact of health care interventions, not only from clinical but also from economic and policy perspectives. Instruments designed to capture HRQoL are essential components of health technology assessment (HTA), where they support cost-utility analyses by enabling the calculation of quality-adjusted life years (QALYs) [1]. These instruments assess various aspects of HRQoL, such as physical and mental functioning, pain, and sleep, which are relevant to both patients and the general population [2]. Among the various tools available, preference-accompanied measures (PAMs) are widely used due to their simplicity, versatility, and the availability of value sets that reflect societal preferences. These instruments provide a standardized framework to quantify HRQoL in a single numeric value (index value), bridging clinical outcomes with decision-making in health economics [3].

Among the widely used PAMs are the EQ-5D, Short Form Survey (SF-6D), Health Utilities Index (HUI), 15D, and Assessment of Quality of Life (AQoL) [4], with the EQ-5D being the most widely adopted globally [5]. More than 20 countries, including Hungary, incorporate the EQ-5D into their HTA guidelines [5–7]. Within the EQ-5D suite of instruments, multiple versions exist to accommodate different age groups and sensitivity needs. For adults, both the EQ-5D-3L (3L) and EQ-5D-5L (5L) are commonly used, with the latter offering greater granularity [8, 9]. To improve suitability for younger populations, the EQ-5D-Y-3L (Y-3L) was introduced for children and adolescents aged 8–15 [10]. While it retains the same five dimensions and three-level structure as the 3L, it uses simplified and age-appropriate wording, such as ‘feeling worried, sad or unhappy’ in place of ‘anxious or depressed.’ The Y-3L has undergone international validation and is gaining traction in youth health research [11–14], although it has not yet been formally validated in Hungary. Conversely, the adult 3L has been thoroughly validated within the Hungarian context across multiple populations and health conditions [15–18]. Hungary has developed value sets for both the 3L and Y-3L, making it possible to conduct locally relevant economic evaluations using either version [19, 20]. Although the EQ-5D-Y-5L (Y-5L) has been introduced internationally as a more detailed alternative to the Y-3L, comparable to the 5L [21], it has not yet been implemented or valued in Hungary, reinforcing the Y-3L’s relevance in the current setting.

The coexistence of instruments across age groups introduces practical challenges in longitudinal health assessments and economic modelling. In HTA practice, distinctions between paediatric and adult populations are not always clear-cut. Treatments for chronic conditions, rare diseases, or preventive interventions such as childhood vaccinations often require HRQoL measurement across the lifespan. Currently, the Y-3L is typically recommended for younger populations, with a switch to the 3L as patients reach adulthood [22]. However, this transition is underexplored [23–25], and discrepancies between instruments may distort long-term evaluations if not adequately accounted for. Index values may differ not only due to true changes in HRQoL but also as a function of the instrument itself, raising concerns about the continuity and comparability of data over time.

Furthermore, although the 3L is designed for adults, its wording and structure may be challenging for certain subgroups, such as individuals with lower literacy or cognitive impairments [26–28]. In such cases, the Y-3L’s simplified language might facilitate more accurate self-reporting, even in adult populations [23]. This highlights the broader relevance of comparing these two descriptive systems in general adult samples, beyond their traditional age-based applications. While the Y-3L was originally developed for younger respondents, its wording may influence how individuals perceive and report health [29], making it important to understand whether the instruments perform differently when used in the same population.

Although some studies have investigated the transition from Y-3L to 3L among adolescents [24, 25], research focusing on adult populations remains scarce, with one study conducted in the United States [23]. However, to our knowledge, no published evidence exists from Central and Eastern Europe. Given the potential impact of cultural and demographic factors on self-reported HRQoL, this study aimed to assess the measurement properties of the Y-3L and 3L in an adult general population sample in Hungary. By evaluating how self-reported HRQoL differs between these instruments, the study contributes to the broader understanding of their performance in adult populations.

## Methods

### Study design and population

This study presents a secondary analysis of cross-sectional data collected during the Hungarian Y-3L valuation study [19, 30]. The sample included adults aged 18 years and older who provided informed consent. Participants were recruited through two survey modes: an online survey administered to members of a large Hungarian online panel (*n* = 996), and computer-assisted personal interviews (CAPIs) conducted by trained interviewers (*n* = 200). To ensure representativeness of the general population, both sampling strategies applied interlocking quotas based on age (18–24, 25–34, 35–44, 45–54, 55–64, 65–74, and 75+) and gender, as defined by census data from the Hungarian Central Statistical Office [31]. Additional ‘soft’ quotas were applied for education, place of residence, and geographical region; these guided recruitment targets were not enforced strictly, allowing some flexibility while aiming for representativeness. Online participants were compensated with redeemable points for gift vouchers or prize draws, whereas CAPI participants received no financial compensation.

Data collection for the online survey took place in April and May 2021, while the CAPIs were conducted between March and September 2021. All participants first completed the Hungarian version of the Y-3L, after which they proceeded to a series of preference elicitation tasks and additional questionnaire items. The 3L was administered afterward, followed by a structured questionnaire that included sociodemographic (gender, age, education, place of residence, geographical region, employment, and marital status) and health-related questions (e.g., self-perceived health, history of physician-diagnosed chronic conditions) in a fixed order across both survey modes. To capture illnesses, participants were asked to report their health conditions and indicate whether they had been diagnosed by a physician with any of 12 common chronic health conditions. Ethical approval for the study was granted by the Research Ethics Committee of the Corvinus University of Budapest (approval no. KRH/31/2021).

### EQ-5D-3L and EQ-5D-Y-3L

Both the 3L and Y-3L instruments are generic PAMs that consist of two components: a five-dimension descriptive system and a visual analogue scale (EQ VAS) [8, 10]. Respondents are asked to evaluate their current health (‘today’) using these measures. In both versions, the EQ VAS allows respondents to rate their health on a vertical scale ranging from 0 (‘the worst health you can imagine’) to 100 (‘the best health you can imagine’).

The 3L assesses five dimensions: mobility, self-care, usual activities, pain/discomfort, and anxiety/depression (the latter phrased as ‘anxiety/feeling down’ in the official Hungarian translation provided by the EuroQol Office). Each dimension is scored on a three-level severity scale, with the responses classified as 1=‘no problems’, 2=‘some/moderate problems’, and 3=‘severe problems/confined to bed’, yielding 3^5^=243 distinct health states [8]. The Y-3L retains the same five dimensions but adapts the wording for age appropriateness [10]. For example, ‘mobility’ is phrased as ‘mobility (walking about)’, while ‘self-care’ is reframed as ‘looking after myself’. Similarly, ‘usual activities’ is adapted to ‘doing usual activities’, and ‘pain/discomfort’ becomes ‘having pain or discomfort’. The ‘anxiety/depression’ dimension is conceptualised as ‘feeling worried, sad, or unhappy’. While the Y-3L maintains the same three-level severity scale, the phrasing is modified for greater accessibility. The response options are worded as 1=‘no problems/no pain or discomfort/not worried, sad or unhappy’, 2=‘some problems/some pain or discomfort/a bit worried, sad or unhappy’, and 3=‘a lot of problems/a lot of pain or discomfort/very worried, sad or unhappy’.

In this study, we applied the Hungarian value sets to derive index values for the 3L and Y-3L [19, 20]. The 3L value set, based on the composite time trade-off (cTTO) method, yields index values ranging from 1 to −0.865. The Y-3L value set, developed using a combination of cTTO and discrete choice experiment (DCE) data, ranges from 1 to −0.485. In both cases, a value of 0 represents a health state equivalent to being dead, while negative values represent states considered worse than dead.

### Statistical analysis

Statistical analyses were conducted using the full sample as well as separately by data collection mode (online vs. CAPI). Descriptive statistics were used to report the distribution of responses for each questionnaire dimension. The relative frequency of responses for each dimension was calculated for the entire sample, categorised by response level. After matching the corresponding dimensions, ceiling and floor were evaluated both at the dimension level and for the overall health profile (i.e., the best and worst possible health states), along with two measures of informativity. To compare the ceiling between the two instruments, McNemar’s test was applied to paired responses for each dimension and for the overall health profile.

Absolute informativity was calculated to measure the diversity of responses within each dimension using Shannon’s index (H’), defined as $\:H{\prime\:}=\:-\sum\:_{i=1}^{L}{p}_{i}*{\mathrm{log}}_{2}{p}_{i}$, where *p*_i_ is the proportion of responses at level *i* (for i = 1, …, L) within a dimension, and L is the number of levels in a dimension (i.e., 3 for the three-level versions). Higher values of H’ indicate greater informational diversity. Relative informativity was measured using Shannon’s Evenness Index (J’) to assess the uniformity of the distribution across levels, computed as $\:{J}^{{\prime\:}}=\frac{H{\prime\:}}{{H{\prime\:}}_{max}}$, where $\:{H{\prime\:}}_{max}={\mathrm{log}}_{2}L$. J’ ranges from 0 (maximum skew) to 1 (perfectly even distribution) [32, 33]. For index values, informativity indices were calculated using distributions binned in intervals of 0.05 to evaluate the spread across the index value scale [34, 35].

In addition to index values, level sum scores (LSS) were calculated by summing the severity levels across the five dimensions. To facilitate interpretation and comparability, LSS values were linearly rescaled to a 0-100 scale, where 0 indicates the best possible health state (full health) and 100 the worst.

To assess agreement between the Y-3L and 3L, cross-tabulations of matched dimensions were conducted. A response pair was classified as inconsistent if the responses to the corresponding dimensions differed [36]. Kendall’s tau correlation coefficients were used to assess the strength of agreement, interpreted as: very weak (< 0.2), weak (0.2–0.39), moderate (0.4–0.59), strong (0.6–0.79), and very strong (≥ 0.8) [37]. Agreement between the Y-3L and 3L was assessed for both index values and LSS using Bland-Altman analysis [38] and the intraclass correlation coefficient (ICC) [39]. A two-way mixed-effects model with absolute agreement was applied [40]. Agreement based on ICC values were interpreted as follows: ICC < 0.4 poor, 0.4 ≤ ICC < 0.6 fair, 0.6 ≤ ICC < 0.75 good, and 0.75 ≤ ICC excellent [41]. Bland-Altman analysis was used to assess agreement between the two instruments by calculating the mean difference and 95% limits of agreement, and by visualizing the distribution of differences.

Known-group validity was examined by comparing the instruments across groups defined by self-perceived health status (5-point excellent-to-poor scale), and presence of physician-diagnosed chronic conditions, with the comparator group consisting of respondents reporting no chronic conditions (i.e., the ‘healthy’ group). Due to the absence of an adult value set for the Y-3L, comparisons were also made using mean LSSs. A sensitivity analysis was performed using index values derived from Hungarian value sets. Differences in LSS and index values were tested using analysis of variance (ANOVA) for self-perceived health and Student’s t-test for chronic conditions compared to the ‘healthy’ group. Additionally, paired-samples t-tests were used to assess whether mean LSS and index values differed significantly between the Y-3L and 3L in the total sample as well as within subgroups. To assess known-group validity, effect size (ES) and relative efficiency (RE) were computed. Eta-squared (η^2^) was calculated for the self-perceived health status, with thresholds for interpretation: negligible (η^2^ < 0.01), small (0.01 ≤ η^2^ < 0.06), medium (0.06 ≤ η^2^ < 0.14), and large (η^2^ ≥ 0.14) [42]. For binary comparisons, Cohen’s d was used, interpreted as: negligible (d < 0.2), small (0.2 ≤ d < 0.5), medium (0.5 ≤ d < 0.8), and large (d ≥ 0.8) [42]. RE was defined as the ratio of the ESs between Y-3L and 3L, using the 3L as reference. An RE > 1 indicated that Y-3L had greater discriminatory ability. Bootstrapping with 2000 samples and accelerated bias correction was used to estimate 95% confidence intervals for RE.

## Results

### Characteristics of the study population

Demographic comparison between the online sample (*n* = 996) and the CAPI sample (*n* = 200) indicated that the two groups were largely similar and representative of the Hungarian population (Table 1). Gender and age distributions did not statistically significantly differ between samples, supporting the decision to pool the data for analysis. However, statistically significant differences were observed for education level, place of residence, geographical region, employment status, and marital status. The online sample had a higher proportion of participants with tertiary education and residents of the capital, but a lower proportion of individuals in domestic partnerships.

**Table 1:** Characteristics of the study population

Variables	Reference population ^a^	Pooled sample (*n* = 1196)		Online sample (*n* = 996)		CAPI sample (*n* = 200)		*p*-value ^b^
	%	*n*	%	*n*	%	*n*	%	
Gender								
Female	53.1	630	52.7	522	52.4	108	54.0	0.681
Male	46.9	566	47.3	474	47.6	92	46.0	
Age, years								
18–24	10.0	125	10.5	103	10.3	22	11.0	0.915
25–34	15.2	188	15.7	157	15.8	31	15.5	
35–44	19.5	234	19.6	195	19.6	39	19.5	
45–54	16.0	198	16.6	167	16.8	31	15.5	
55–64	16.8	205	17.1	172	17.3	33	16.5	
65–74	13.0	159	13.3	134	13.5	25	12.5	
75+	9.6	87	7.3	68	6.8	19	9.5	
Highest level of education								
Primary school or less	45.4	276	23.1	219	22.0	57	28.5	0.013
Secondary school ^c^	33.3	448	37.5	366	36.7	82	41.0	
College/university degree	21.3	472	39.5	411	41.3	61	30.5	
Place of residence								
Capital	17.9	318	26.6	276	27.7	42	21.0	0.005
Other town	52.6	608	50.8	512	51.4	96	48.0	
Village	29.5	270	22.6	208	20.9	62	31.0	
Geographical region								
Central Hungary	30.4	427	35.7	378	38.0	49	24.5	< 0.001
Western Hungary	30.2	357	29.8	272	27.3	85	42.5	
Eastern Hungary	39.5	412	34.4	346	34.7	66	33.0	
Employment status ^d^								
Employed	53.1	691	57.8	572	57.4	119	59.5	0.026
Retired	26.1	281	23.5	228	22.9	53	26.5	
Disability pensioner	3.1	28	2.3	22	2.2	6	3.0	
Student	3.1	91	7.6	74	7.4	17	8.5	
Unemployed	4.7	61	5.1	59	5.9	2	1.0	
Homemaker/housewife	1.0	44	3.7	41	4.1	3	1.5	
Marital status								
Married	45.6	484	40.5	410	41.2	74	37.0	0.007
Domestic partnership	13.4	236	19.7	181	18.2	55	27.5	
Single	18.5	259	21.7	222	22.3	37	18.5	
Widowed	11.4	95	7.9	74	7.4	21	10.5	
Divorced	11.1	96	8.0	88	8.8	8	4.0	
Other	–	26	2.2	21	2.1	5	2.5	
Self-perceived health status								
Excellent	NA	102	8.5	67	6.7	35	17.5	< 0.001
Very good	NA	321	26.8	252	25.3	69	34.5	
Good	NA	489	40.9	432	43.4	57	28.5	
Fair	NA	244	20.4	212	21.3	32	16.0	
Poor	NA	40	3.3	33	3.3	7	3.5	
History of chronic illness ^e, f^								
Yes	48.0	777	65.0	666	66.9	111	55.5	< 0.001
No	52.0	400	33.4	312	31.3	88	44.0	

CAPI computer-assisted personal interview, NA not available

^a^ Hungarian Central Statistical Office Microcensus 2016 (1)

^b^ Pearson’s χ^2^ test was used to assess the difference in relative frequency between the responses of the online and CAPI samples

^c^ With completed final examination

^d^ The sum of the general population is < 100% owing to an ‘other’ category accounting for 8.9%

^e^ *N* = 19 refused to answer

^f^ Reference values: European Health Interview Survey 2019 (2)

### Dimension-level analysis

Across dimensions, ceiling was the highest for looking after myself vs. self-care (95.2% in Y-3L; 95.5% in 3L) and lowest for pain/discomfort (56.4% in Y-3L; 62.0% in 3L) (Table 2). The 3L consistently showed higher ceiling across most dimensions, particularly for anxiety/depression vs. worried/sad/unhappy, where 71.6% of respondents reported no problems compared to 56.8% in the Y-3L. All differences in ceiling between versions were statistically significant except for looking after myself vs. self-care. While floor was low overall, the Y-3L showed notably higher floor compared to the 3L, particularly in mobility (5.4% in Y-3L; 0.3% in 3L). Relative informativity (J’) was consistently higher for the Y-3L (range: 0.20–0.75) than for the 3L dimensions (range: 0.18–0.66) (Table 2). The average J’ also was improved for the Y-3L (0.56) vs. 3L (0.48).

**Table 2:** Ceiling, floor, informativity and agreement (pooled sample, *n* = 1196)

Dimensions	Level 1 (ceiling) *n* (%)				Level 2 *n* (%)		Level 3 (floor) *n* (%)				Absolute informativity (H’)		Relative informativity (J’)		Agreement (Kendall’s tau)
	EQ-5D-Y-3L	EQ-5D-3L	Absolute ceiling difference (pp)	Relative ceiling difference (%)	EQ-5D-Y-3L	EQ-5D-3L	EQ-5D-Y-3L	EQ-5D-3L	Absolute floor difference (pp)	Relative floor difference (%)	EQ-5D-Y-3L	EQ-5D-3L	EQ-5D-Y-3L	EQ-5D-3L	
Mobility ^a,^ ^b^	874 (73.1)	893 (74.7)	1.6	2.1	257 (21.5)	299 (25.0)	65 (5.4)	4 (0.3)	−5.1	−1525.0	1.04	0.84	0.65	0.53	0.841
Looking after myself/Self-care ^b^	1139 (95.2)	1142 (95.5)	0.3	0.3	44 (3.7)	51 (4.3)	13 (1.1)	3 (0.3)	−0.8	−333.3	0.31	0.28	0.20	0.18	0.745
Doing usual activities/Usual activities ^a,^ ^b^	991 (82.9)	1014 (84.8)	1.9	2.3	177 (14.8)	176 (14.7)	28 (2.3)	6 (0.5)	−1.8	−366.7	0.76	0.65	0.48	0.41	0.721
Having pain/discomfort/Pain/discomfort ^a,^ ^b^	675 (56.4)	741 (62.0)	5.6	8.9	491 (41.1)	437 (36.5)	30 (2.5)	18 (1.5)	−1.0	−66.7	1.13	1.05	0.71	0.66	0.764
Feeling worried, sad or unhappy/Anxiety/depression ^a, b^	679 (56.8)	856 (71.6)	14.8	20.7	466 (39.0)	318 (26.6)	51 (4.3)	22 (1.8)	−2.5	−131.8	1.19	0.96	0.75	0.61	0.636
Total/average ^a^	416 (34.8)	560 (46.8)	12.0	25.7	-	-	0 (0.0)	0 (0.0)	-	-	0.88	0.76	0.56	0.48	-

McNemar’s test was used to assess the difference in ceiling and floor between EQ-5D-Y-3L and EQ-5D-3L. Statistically significant differences in ceiling are marked with superscript ‘a’ and differences in floor with superscript ‘b’ (*p* < 0.05)

Agreement between corresponding dimensions, assessed using Kendall’s tau, was strong/very strong across all dimensions (range: 0.636–0.841), with the highest agreement observed in mobility and the lowest in anxiety/depression vs. worried/sad/unhappy (Table 2). The cross-tabulation of responses between the Y-3L and 3L also revealed a high degree of agreement across most dimensions (Table 3). Overall, 89.2% of responses in the mobility dimension were consistent between the two instruments. The consistency was notably higher in the looking after myself vs. self-care pair, with 97.0% of responses consistent between the two versions. Among respondents who reported some problems washing or dressing on the 3L, 23.5% reported no problems on the Y-3L. Other dimensions, such as usual activities and pain/discomfort, exhibited strong consistency as well, with rates of 90.8% and 87.1%, respectively. For usual activities, 19.3% of those reporting some problems with performing usual activities on the 3L reported to have no problems doing their usual activities on the Y-3L. Regarding pain/discomfort, 13.6% of those reporting to have no pain or discomfort on the 3L reported at least some pain or discomfort on the Y-3L. Nonetheless, the worried/sad/unhappy vs. anxiety/depression pair showed the lowest consistency across instruments, with 77.9%. Notably, 22.9% of those reporting not being anxious or depressed on the 3L reported to be at least a bit worried, sad or unhappy on the Y-3L. Furthermore, 31.8% of those being extremely anxious or depressed were only a bit worried, sad or unhappy.

**Table 3:** Cross-tabulation of EQ-5D-Y-3L and EQ-5D-3L responses (pooled sample, *n* = 1196)

EQ-5D-Y-3L	EQ-5D-3L			Consistent response pairs (*n*, %)
Mobility (Y-3L) vs. Mobility (3L), n (%)	*I have* *no* *problems walking about*	*I have* *some* *problems walking about*	*I am* *confined to bed*	1067 (89.2)
* I have* *no* *problems walking about*	850 (95.2)	24 (8.0)	0 (0.0)	
* I have* *some* *problems walking about*	42 (4.7)	214 (71.6)	1 (25.0)	
* I have* *a lot* *of problems walking about*	1 (0.1)	61 (20.4)	3 (75.0)	
Looking after myself (Y-3L) vs. Self-care (3L), n (%)	*I have* *no problems with self-care*	*I have* *some* *problems washing or dressing myself*	*I am* *unable to* *wash or dress myself*	1160 (97.0)
* I have* *no* *problems washing or dressing myself*	1127 (98.7)	12 (23.5)	0 (0.0)	
* I have* *some* *problems washing or dressing myself*	14 (1.2)	30 (58.8)	0 (0.0)	
* I have* *a lot* *of problems washing or dressing myself*	1 (0.1)	9 (17.6)	3 (100.0)	
Doing usual activities (Y-3L) vs. Usual activities (3L), n (%)	*I have* *no* *problems with performing my usual activities*	*I have* *some* *problems with performing my usual activities*	*I am* *unable to* *perform my usual activities*	1086 (90.8)
* I have* *no* *problems doing my usual activities*	957 (94.4)	34 (19.3)	0 (0.0)	
* I have* *some* *problems doing my usual activities*	54 (5.3)	123 (69.9)	0 (0.0)	
* I have* *a lot* *of problems doing my usual activities*	3 (0.3)	19 (10.8)	6 (100.0)	
Having pain/discomfort (Y-3L) vs. Pain/discomfort (3L), n (%)	*I have* *no* *pain or discomfort*	*I have* *moderate* *pain or discomfort*^*1*^	*I have* *extreme* *pain or discomfort*^*2*^	1042 (87.1)
* I have* *no* *pain or discomfort*	638 (86.1)	37 (8.5)	0 (0.0)	
* I have* *some* *pain or discomfort*	101 (13.6)	388 (88.8)	2 (11.1)	
* I have* *a lot* *of pain or discomfort*	2 (0.3)	12 (2.7)	16 (88.9)	
Feeling worried, sad or unhappy (Y-3L) vs. Anxiety/depression (3L), n (%)	*I am* *not* *anxious or depressed*	*I am* *moderately* *anxious or depressed*^*3*^	*I am* *extremely* *anxious or depressed*^*4*^	932 (77.9)
* I am* *not worried*,* sad or unhappy*	654 (76.4)	25 (7.9)	0 (0.0)	
* I am* *a bit* *worried*,* sad or unhappy*	196 (22.9)	263 (82.7)	7 (31.8)	
* I am* *very* *worried*,* sad or unhappy*	6 (0.7)	30 (9.4)	15 (68.2)	

Percentages may not total 100 by rows due to rounding

1-in Hungarian: I have moderate pain or a little discomfort

2-in Hungarian: I have very strong pain or very large discomfort

3-in Hungarian: I am moderately anxious or feeling down a little

4-in Hungarian: I am very much anxious or feeling down a lot

### Analysis of the level sum scores and index values

Although both instruments theoretically define 243 health states, in our sample more were observed in the Y-3L (n = 85) than in the 3L (n = 47), representing 35.0% vs. 19.3% of all possible health state profiles, respectively (Table 4). The proportion of consistent health state profiles across both measures was 59.0% including full health (‘11111’), but only 26.1% when full health was excluded. This was reflected in overall ceiling, with 34.8% of Y-3L and 46.8% of 3L respondents reporting full health. No participants reported the worst possible health state in either instrument. J’ for index value distributions (binned in intervals of 0.05) was also higher in the Y-3L (0.47) than in the 3L (0.40).

**Table 4:** Characteristics of the EQ-5D-Y-3L and EQ-5D-3L health state profiles (pooled sample, *n* = 1196)

	EQ-5D-Y-3L			EQ-5D-3L		
Theoretical number of health state profiles	243			243		
Observed health state profiles	85			47		
Proportion of health state profiles used (%*)*	35.0			19.3		
Respondents with consistent health profiles (*including* ‘11111’) (n, %)	706 (59.0)					
Respondents with consistent health profiles (*excluding* ‘11111’) (n, %)	312 (26.1)					
Intraclass correlation coefficient for level sum scores (95% CI)	0.811 (0.734–0.860)					
Intraclass correlation coefficient for index values (95% CI)	0.735 (0.649–0.795)					
Floor (%)	0			0		
Ceiling (%)	34.8			46.8		
Shannon’s index (H’)	3.75			3.13		
H’ max	7.92			7.92		
Shannon’s evenness index (J’)	0.47			0.40		
10 most common health state profiles	Profile	Frequency	Relative frequency (%)	Profile	Frequency	Relative frequency (%)
	11111	416	34.8	11111	560	46.8
	11112	162	13.5	11122	104	8.7
	11122	141	11.8	11121	82	6.9
	11121	78	6.5	11112	82	6.9
	21121	52	4.3	21121	68	5.7
	21122	42	3.5	21111	53	4.4
	21111	37	3.1	21122	37	3.1
	21222	35	2.9	21221	33	2.8
	21221	18	1.5	21222	28	2.3
	11222	17	1.4	11222	24	2.0

Intraclass correlation coefficients (ICC) were calculated using a two-way mixed-effects model with absolute agreement

**Fig. 1:**
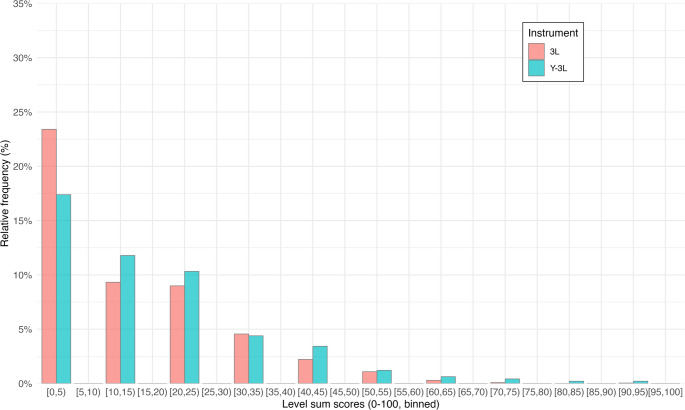
Distribution of respondents across level sum scores on the EQ-5D-3L and EQ-5D-Y-3L (pooled sample, *n* = 1196)

**Fig. 2:**
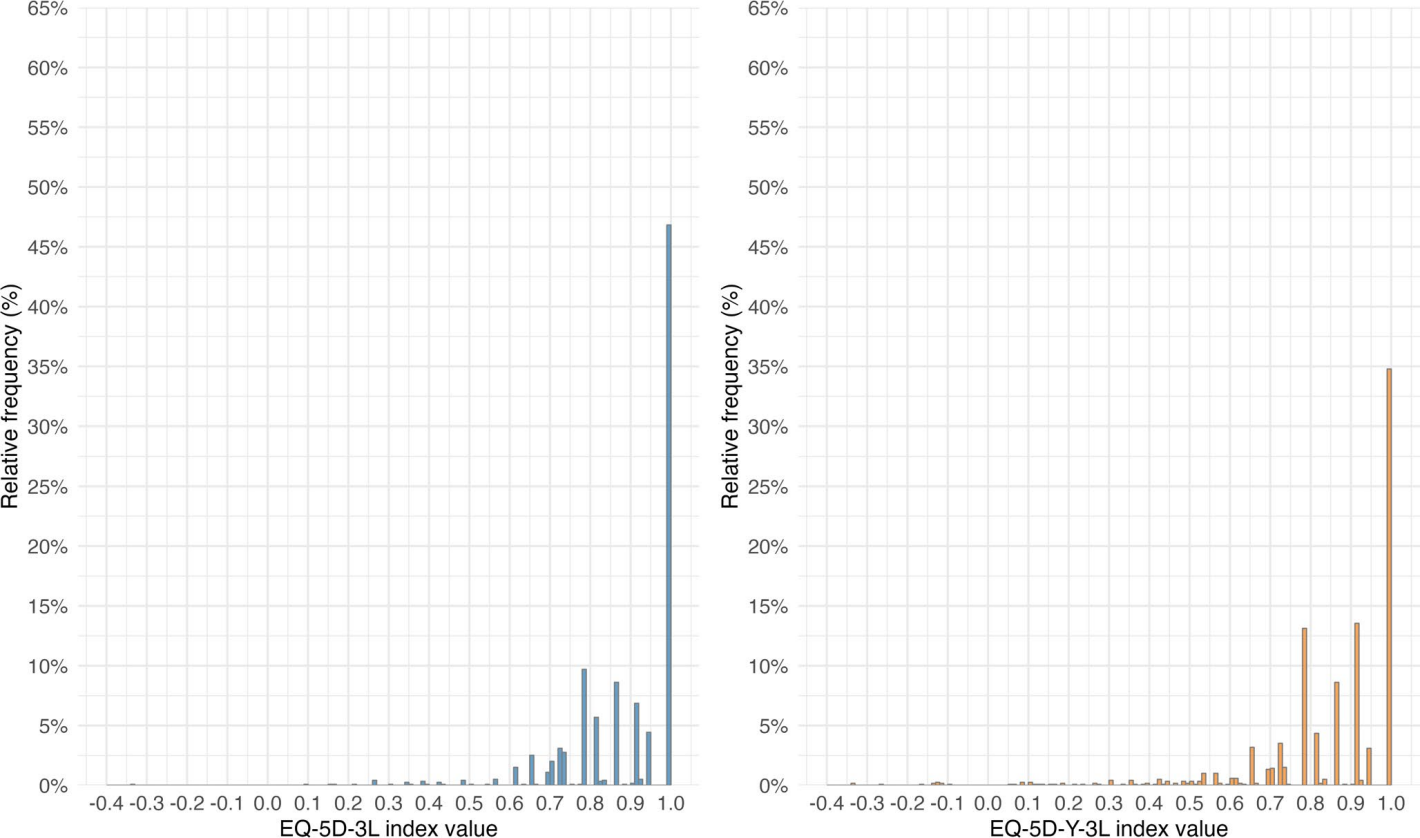
Distribution of respondents across index values on the EQ-5D-3L and EQ-5D-Y-3L (pooled sample, *n* = 1196)

Figures 1 and 2 show the distributions of LSS and index values for the Y-3L and 3L. In both cases, the 3L exhibited a pronounced ceiling, with a high concentration of respondents reporting full health. The Y-3L distributions were more dispersed, with lower average scores and greater use of mid-range values and a higher frequency of level 3 responses. These patterns were consistent across LSS and index values.

**Fig. 3:**
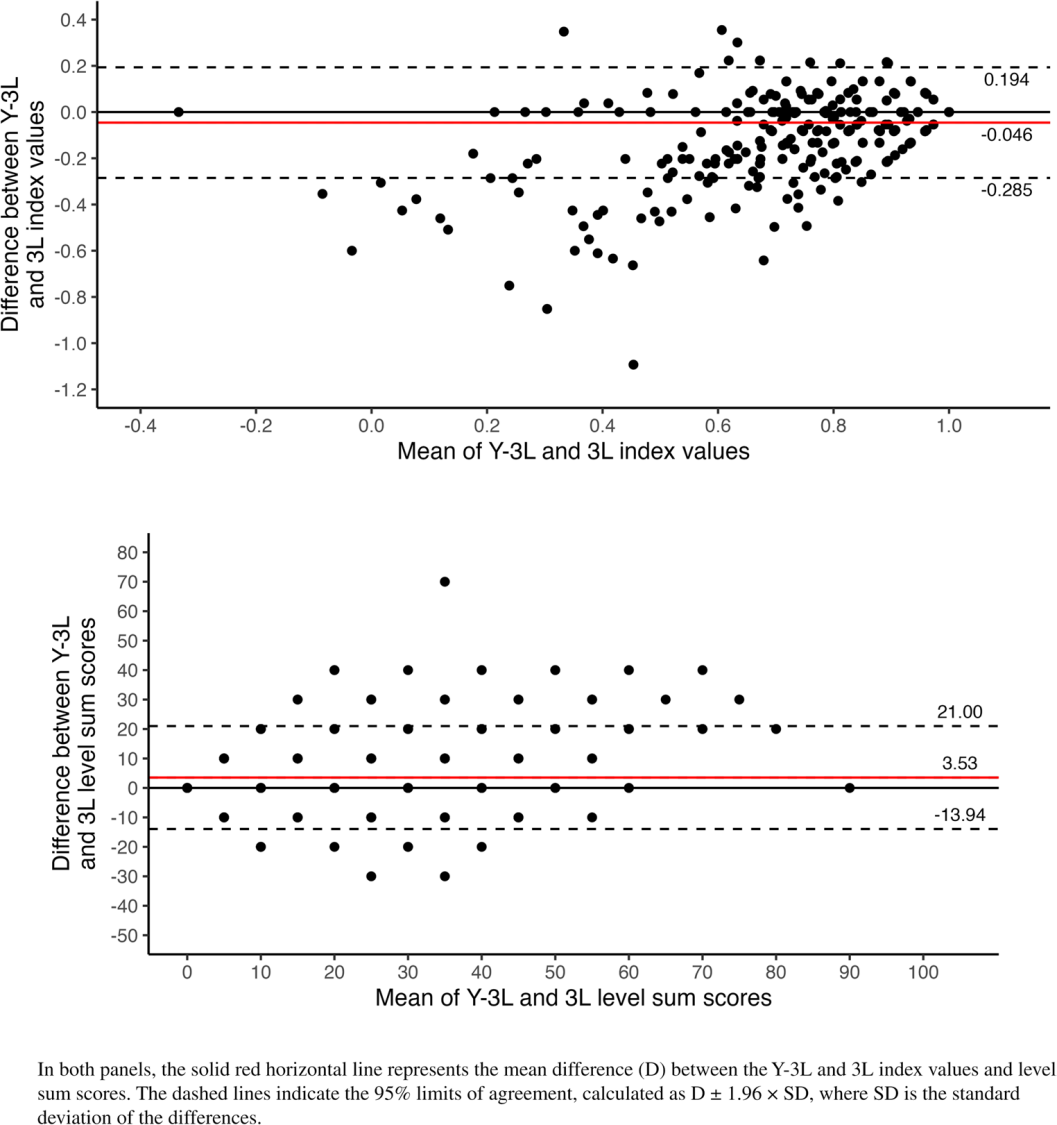
Bland-Altman plots comparing Y-3L and 3L index values and level sum scores (pooled sample, *n* = 1196)

Results of the Bland-Altman analysis for index values and level sum scores are presented in Fig. 3. For index values, the mean difference was − 0.046, with 95% limits of agreement from − 0.285 to 0.194, indicating slightly lower values on the Y-3L. For level sum scores, the mean difference was 3.53 (limits of agreement: −13.94 to 21.00), suggesting that Y-3L tended to capture more problems. Discrepancies were most pronounced among respondents in worse health states.

The mean LSS was significantly higher on the Y-3L than on the 3L in the total sample, at 15.13 and 11.60, respectively. This pattern was consistent across all self-perceived health categories and chronic health conditions (Table 5). A clear gradient emerged, with LSS increasing as self-perceived health deteriorated. Similarly, respondents with chronic health conditions consistently had higher LSS compared to healthy individuals. Paired-samples t-tests showed significantly higher LSS on the Y-3L than the 3L across all known groups, except for anxiety. Both instruments showed large effect sizes for self-perceived health (Y-3L: 0.333; 3L: 0.332) and across all chronic condition groups (Y-3L: 0.820–2.454; 3L: 0.820–2.696), supporting strong known-groups validity. Overall, the Y-3L proved to be more efficient in distinguishing between 8/13 known groups (RE > 1). However, bootstrap confidence intervals indicated statistically significant differences only for anxiety (RE = 0.844), where the 3L was more efficient. A similar trend was observed in the index values (Table 6). In the total sample, the mean index value was lower on the Y-3L than on the 3L, reaching 0.842 and 0.887, respectively. Index values were significantly lower on the Y-3L than the 3L in all subgroups, with values declining alongside worsening self-perceived health and in the presence of chronic conditions. Medium and large ESs were observed for self-perceived health categories (Y-3L: 0.302; 3L: 0.311) and across all chronic condition groups (Y-3L: 0.754–2.362; 3L: 0.747–2.365). The Y-3L showed higher RE in 9/13 comparisons, but none reached statistical significance.

**Table 5:** Known-groups validity of level sum scores (0-100) (pooled sample, *n* = 1196)

	*n* (%)	EQ-5D-Y-3L			EQ-5D-3L			Y-3L vs. 3L paired t-test (*p*-value)	RE ^d^	95% CI ^e^
		Mean (SD)	*p*-value	ES ^c^	Mean (SD)	*p*-value	ES ^c^			
Total sample	1196 (100.0)	15.13 (16.54)	-	-	11.60 (14.13)	-	-	< 0.001	-	-
Self-perceived health status ^a^										
Excellent	102 (8.5)	3.63 (8.42)	< 0.001	0.333	1.18 (3.53)	< 0.001	0.332	0.002	1.004	0.892-1.100
Very good	321 (26.8)	6.67 (9.28)			4.45 (7.69)			< 0.001		
Good	489 (40.9)	14.00 (12.50)			10.70 (11.80)			< 0.001		
Fair	244 (20.4)	27.40 (17.00)			22.80 (14.70)			< 0.001		
Poor	40 (3.3)	51.20 (23.10)			38.80 (16.50)			< 0.001		
Health conditions ^b^										
Healthy	400 (33.5)	7.38 (9.52)	-	-	5.00 (8.64)	-	-	< 0.001	-	
Allergies	180 (15.1)	17.72 (17.68)	< 0.001	0.820	14.33 (15.86)	< 0.001	0.820	< 0.001	0.999	0.883–1.143
Skin disease	88 (7.4)	18.07 (16.81)	< 0.001	0.957	14.55 (15.16)	< 0.001	0.943	< 0.001	1.014	0.888–1.231
Hypertension	360 (30.1)	21.36 (19.72)	< 0.001	0.918	16.75 (16.48)	< 0.001	0.907	< 0.001	1.013	0.947–1.092
Asthma, COPD	65 (5.4)	21.08 (18.55)	< 0.001	1.222	18.31 (17.19)	< 0.001	1.297	0.017	0.942	0.807–1.083
Gastrointestinal disease	88 (7.4)	22.27 (18.68)	< 0.001	1.274	18.30 (17.24)	< 0.001	1.243	0.006	1.025	0.868–1.226
Cancer	41 (3.4)	25.12 (14.16)	< 0.001	1.769	21.46 (14.24)	< 0.001	1.772	0.014	0.999	0.838–1.165
Diabetes	118 (9.9)	25.17 (22.41)	< 0.001	1.312	19.32 (18.25)	< 0.001	1.241	< 0.001	1.058	0.952–1.197
Osteoporosis	37 (3.1)	25.68 (21.54)	< 0.001	1.660	20.00 (13.94)	< 0.001	1.631	0.008	1.018	0.850–1.236
Cardiovascular disease	139 (11.6)	28.56 (23.64)	< 0.001	1.459	22.16 (18.76)	< 0.001	1.420	< 0.001	1.027	0.948–1.134
Musculoskeletal diseases	279 (23.3)	28.50 (19.30)	< 0.001	1.470	22.72 (15.77)	< 0.001	1.466	< 0.001	1.003	0.940–1.081
*** Anxiety***	***81 (6.8)***	***31.36 (21.38)***	***< 0.001***	***1.947***	***29.01 (16.63)***	***< 0.001***	***2.306***	***0.102***	***0.844***	***0.758–0.927***
Depression	56 (4.7)	36.07 (21.72)	< 0.001	2.454	31.79 (16.53)	< 0.001	2.696	0.018	0.910	0.809–1.016

COPD chronic obstructive pulmonary disease, ES effect size, RE relative efficiency

Bold italic rows indicate statistically significant differences in relative efficiency (RE), where one instrument performed significantly better (95% CI does not include 1)

^a^ Analysis of variance to test the difference between categories, where *p* < 0.05 was considered statistically significant

^b^ Student’s t-test compared to the healthy subgroup, where *p* < 0.05 was considered statistically significant

^c^ Effect sizes were calculated using Cohen’s d (two groups) or η^2^ (three or more groups)

^d^ Relative efficiency compared to EQ-5D-3L

^e^ 2000 bootstrap samples with accelerated bias correction

**Table 6:** Known-groups validity of index values (pooled sample, *n* = 1196)

	*n* (%)	EQ-5D-Y-3L			EQ-5D-3L			Y-3L vs. 3L paired t-test (*p*-value)	RE ^d^	95% CI ^e^
		Mean (SD)	*p*-value	ES ^c^	Mean (SD)	*p*-value	ES ^c^			
Total sample	1196 (100.0)	0.842 (0.201)	-	-	0.887 (0.148)	-	-	< 0.001	-	-
Self-perceived health status ^a^										
Excellent	102 (8.5)	0.962 (0.116)	< 0.001	0.302	0.988 (0.037)	< 0.001	0.311	0.021	0.972	0.806–1.077
Very good	321 (26.8)	0.934 (0.097)			0.958 (0.075)			< 0.001		
Good	489 (40.9)	0.863 (0.129)			0.899 (0.110)			< 0.001		
Fair	244 (20.4)	0.708 (0.221)			0.779 (0.160)			< 0.001		
Poor	40 (3.3)	0.347 (0.365)			0.580 (0.253)			< 0.001		
Health conditions ^b^										
Healthy	400 (33.5)	0.926 (0.103)	-	-	0.950 (0.093)	-	-	< 0.001	-	-
Allergies	180 (15.1)	0.817 (0.209)	< 0.001	0.754	0.861 (0.164)	< 0.001	0.747	< 0.001	1.010	0.887–1.151
Skin disease	88 (7.4)	0.812 (0.190)	< 0.001	0.922	0.859 (0.141)	< 0.001	0.882	< 0.001	1.046	0.882–1.283
Hypertension	360 (30.1)	0.771 (0.255)	< 0.001	0.813	0.837 (0.181)	< 0.001	0.799	< 0.001	1.017	0.946–1.105
Asthma, COPD	65 (5.4)	0.775 (0.239)	< 0.001	1.154	0.814 (0.210)	< 0.001	1.166	0.024	0.990	0.810–1.173
Gastrointestinal disease	88 (7.4)	0.755 (0.242)	< 0.001	1.233	0.812 (0.201)	< 0.001	1.152	0.005	1.070	0.900-1.294
Cancer	41 (3.4)	0.754 (0.160)	< 0.001	1.566	0.800 (0.157)	< 0.001	1.496	0.015	1.047	0.852–1.334
Diabetes	118 (9.9)	0.717 (0.305)	< 0.001	1.222	0.810 (0.204)	< 0.001	1.098	< 0.001	1.113	0.993–1.270
Osteoporosis	37 (3.1)	0.722 (0.280)	< 0.001	1.599	0.806 (0.146)	< 0.001	1.469	0.025	1.088	0.861–1.450
Cardiovascular disease	139 (11.6)	0.669 (0.320)	< 0.001	1.388	0.777 (0.215)	< 0.001	1.275	< 0.001	1.089	0.994–1.199
Musculoskeletal diseases	279 (23.3)	0.695 (0.261)	< 0.001	1.252	0.784 (0.177)	< 0.001	1.239	< 0.001	1.010	0.936–1.098
Anxiety	81 (6.8)	0.646 (0.293)	< 0.001	1.839	0.710 (0.203	< 0.001	2.026	0.007	0.908	0.805–1.040
Depression	56 (4.7)	0.592 (0.297)	< 0.001	2.362	0.682 (0.210)	< 0.001	2.365	0.003	0.999	0.857–1.170

COPD chronic obstructive pulmonary disease, ES effect size, RE relative efficiency

^a^ Analysis of variance to test the difference between categories, where *p* < 0.05 was considered statistically significant

^b^ Student’s t-test compared to the healthy subgroup, where *p* < 0.05 was considered statistically significant

^c^ Effect sizes were calculated using Cohen’s d (two groups) or η^2^ (three or more groups)

^d^ Relative efficiency compared to EQ-5D-3L

^e^ 2000 bootstrap samples with accelerated bias correction

### Subgroup analysis by mode of administration

Subgroup analyses were conducted separately for the online and CAPI samples to assess how the Y-3L and 3L performed within each mode of administration. In both samples, ceiling was consistently lower for the Y-3L than for the 3L. In the online group, the ceiling was 31.2% on the Y-3L and 44.1% on the 3L. In the CAPI sample, these figures were 52.5% and 60.5%, respectively. The most notable ceiling differences were observed in the anxiety/depression vs. worried/sad/unhappy dimension, where the Y-3L consistently yielded lower ceilings than the 3L in both modes (53.5% vs. 68.9% online; 73.0% vs. 85.0% CAPI) (Online resources 1–6).

Agreement between instruments remained strong within both subsamples. Kendall’s tau coefficients were ranged from 0.654 (anxiety/depression vs. worried/sad/unhappy) to 0.864 (mobility) in the CAPI sample, and from 0.627 to 0.835 in the online sample. Dimensional agreement was lowest in the anxiety/depression vs. worried/sad/unhappy pair in both subgroups, again showing the lowest consistency (76.3% online; 86.0% in CAPI). (Online resources 7–8).

Relative informativity was higher for the Y-3L than the 3L in both modes of administration. In the online sample, average J’ was 0.56 for the Y-3L and 0.48 for the 3L. In the CAPI sample, the corresponding values were 0.51 and 0.42, respectively (Online resources 5–6). Additionally, the Y-3L produced a greater number of unique health state profiles in both modes (76 vs. 38 online; 35 vs. 26 CAPI) (Online resources 9–10).

Bland-Altman plots for the online and CAPI subsamples confirmed that Y-3L index values were generally lower and LSS values higher than those of the 3L, with wider variation at poorer health levels (Online resources 11–12).

Known-groups validity was supported across both data collection modes. ESs were large in nearly all comparisons. In the online sample, the Y-3L showed higher RE than the 3L in 5/13 known groups based on LSS and in 7/13 based on index values. In the CAPI sample, the Y-3L outperformed the 3L in 9/13 comparisons based on both LSS and index values. However, statistically significant differences in RE were observed in only two cases in the online sample: for LSS, the 3L was more efficient in the anxiety group (RE = 0.833), while for index values, the Y-3L outperformed the 3L in the diabetes group (RE = 1.154). In the CAPI sample, no RE differences reached statistical significance (Online resources 13–16).

## Discussion

This study assessed the differences in self-reported HRQoL and measurement performance of the Y-3L and 3L in a representative sample of Hungarian adults. The two instruments showed substantial agreement across most dimensions, particularly in mobility and self-care, and both demonstrated consistent known-groups validity. However, notable discrepancies emerged in the mental health dimension, where the Y-3L’s ‘feeling worried, sad or unhappy’ captured a broader range of responses than the 3L’s ‘anxiety/depression.’ The Y-3L also produced nearly twice as many unique health state profiles as the 3L, indicating greater response variation. While over half of the participants reported identical health profiles across instruments, the Y-3L exhibited a significantly lower ceiling, especially in the mental health dimension, with fewer respondents reporting no problems. Relative informativity was also higher for the Y-3L across all dimensions. Both instruments effectively distinguished between self-perceived health status and chronic condition subgroups, with the Y-3L showing slightly larger effect sizes in 62–69% of comparisons. These patterns were consistent across administration modes, with both the Y-3L and 3L demonstrating similar discriminatory performance within the online and CAPI samples.

Our findings are consistent with previous research indicating that the Y-3L is able to capture more reported problems and shows a lower prevalence of full health compared to the 3L. In a U.S. general population sample of adults, full health was reported by 33.9% on the Y-3L and 43.6% on the 3L, with increased reporting in anxiety/depression vs. worried/sad/unhappy dimension contributing to this difference [23]. Specifically, 12.5% of respondents who reported no problems in the anxiety/depression dimension on the 3L reported to be a bit worried, sad or unhappy on the Y-3L. This aligns with our results, where full health was reported by 34.8% on the Y-3L and 46.8% on the 3L. Similar patterns were reported in adolescent general population samples [24, 25]. In a South African study (administered in English), full health was reported by 45.0% of adolescents on 3L and 37.2% on the Y-3L. The largest discrepancy occurred in the anxiety/depression dimension, the ceiling dropped from 70.8% (3L) to 60.4% (Y-3L), closely reflecting our adult findings. That study also found higher relative informativity for the Y-3L in most dimensions (Y-3L: 0.22–0.83; 3L: 0.25–0.80), consistent with our results (Y-3L: 0.20–0.75; 3L: 0.18–0.66). In a Chinese adolescent sample (administered in Chinese), the proportion reporting full health was 78.2% on the 3 L and 66.0% on the Y-3L, and the ceiling in the anxiety/depression dimension dropped from 82.9% (3L) to 73.6% (Y-3L). That study also found higher relative informativity for the Y-3L across all dimensions (Y-3L: 0.37–0.72; 3L: 0.31–0.66). Although these two studies focused on adolescents, our findings extend the same pattern to a general adult population. The consistency across age groups and cultural settings suggests that the differences between the Y-3L and 3L are not solely age-related but are likely driven by linguistic characteristics of the instruments themselves. These results reinforce the potential of the Y-3L to capture subtler impairments in HRQoL.

A key consideration when transitioning between HRQoL instruments is conceptual equivalence, ensuring that different instruments measure the same underlying construct despite differences in wording. While both the Y-3L and 3L assess mental health, their language differs: the 3L uses ‘anxiety/depression’, whereas the Y-3L uses ‘feeling worried, sad, or unhappy’, a phrasing originally designed for children and adolescents. Notably, the Hungarian 3L version uses ‘anxiety/feeling down,’ which may already reflect a less clinical tone than the English source version. Although these adaptations improve accessibility, they may also influence how respondents interpret and report their mental states. Our findings suggest that the Y-3L’s simpler wording may capture a broader spectrum of mental health problems. These differences could encourage the reporting of mild or moderate problems that might go unreported using more clinical language in the 3L. Interestingly, newer instruments such as the EQ Health and Wellbeing questionnaire also include items, such as ‘sad’ and ‘depressed’, signalling a potentially broader trend toward capturing a wider range of mental health issues [43]. In this context, the Y-3L wording may be particularly suitable for general population assessments, where simplified phrasing can make it easier for respondents to express mental health-related problems. However, it is important to recognise that feeling sad and being clinically depressed are not interchangeable. While broader terminology may improve sensitivity to milder issues, more specific wording such as ‘being depressed’ may prove to be more appropriate for clinical settings [44]. Additionally, other dimensions may also be affected by wording differences. For example, in the mobility dimension, level 3 is described as ‘confined to bed’ in the 3L but as ‘a lot of problems walking about’ in the Y-3L, which may lead to higher endorsement rates due to the less extreme phrasing.

Several limitations should be acknowledged. First, data were collected using a dual-mode design (online and CAPI), which could introduce mode-related bias. However, analyses conducted within each subsample revealed largely consistent results; therefore, combining the samples for pooled analysis was deemed appropriate. Nevertheless, potential residual mode effects cannot be entirely ruled out. Second, the fixed administration order (Y-3L followed by preference elicitation tasks and then the 3L) may have introduced order effects. Although previous research suggests that the impact of fixed questionnaire order is generally negligible in the context of longer surveys [45–47], the inclusion of preference elicitation tasks between the two instruments may have further sensitized respondents to health-related concepts, potentially influencing how they interpreted and responded to 3L dimensions. Third, the absence of an adult-specific value set for the Y-3L necessitated the use of LSS rather than index values in some analyses, as the available Hungarian Y-3L value set reflects preferences for a hypothetical 10-year-old child. The LSS approach assumes equal importance across all dimensions and does not apply preference or non-preference weights. As a result, it may overestimate or underestimate true differences in HRQoL, depending on which dimensions drive the variation. Nevertheless, its robust performance has been demonstrated in methodological research [48]. Fourth, data collection took place during the COVID-19 pandemic, which may have influenced responses, particularly in the mental health dimensions [49, 50]. Finally, while transitions between the Y-3L and 3L most commonly occur during adolescence, this study focused on adults. Nevertheless, the findings remain relevant for informing such transitions, as well as for evaluating the broader applicability of the Y-3L in adult populations, particularly among subgroups for whom simplified language may enhance response accuracy.

Future research may aim to better align the Y-3L and 3L to ensure consistency in HRQoL assessments across the lifespan. Investigating the feasibility of using the Y-3L in selected adult populations, such as those with lower educational attainment or cognitive limitations, may also be valuable. Research could further examine whether agreement between the Y-3L and 3L differs by respondent characteristics, particularly in transitional age groups or among individuals with lower levels of literacy. Developing crosswalk algorithms could help address discrepancies when transitioning between these instruments. Finally, longitudinal studies following individuals as they transition from the Y-3L to the 3L, or from the Y-5L to the 5L, are especially needed to evaluate consistency over time and mitigate artificial shifts in reported HRQoL.

## Conclusion

This study highlights key differences in self-reporting HRQoL and measurement properties of the EQ-5D-Y-3L and EQ-5D-3L in the general adult population. While the instruments showed strong overall agreement, notable discrepancies in response patterns—particularly in feeling worried, sad, or unhappy vs. anxiety/depression—suggest that transitions should be approached with caution. The EQ-5D-Y-3L’s greater informativity and lower ceiling point to potential utility beyond paediatric populations; however, its use in adults warrants careful consideration, especially in contexts requiring consistency with the 3L. Future research may focus on developments to better aligning the two instruments to support coherent HRQoL measurement across the lifespan.

## Supplementary Information

Below is the link to the electronic supplementary material.

Supplementary Material 1 (DOCX 924 KB)

## Data Availability

All data in this study are available from Fanni Rencz upon reasonable request.

## Code Availability

The code used for the statistical analysis is available from the corresponding author upon reasonable request.
