# Supplementary material for: Comparing the measurement properties of the EQ-5D-Y-3L and EQ-5D-3L in a general population sample of adults
Source: Eur J Health Econ. 2025 Oct 17;27(3):675–89. doi: 10.1007/s10198-025-01839-7 (PMC13190750; doi:10.1007/s10198-025-01839-7)
Supplement: Supplementary file 1 — Supplementary Material 1 (DOCX 924 KB) [file 10198_2025_1839_MOESM1_ESM.docx]

Online resource 1 Distribution of respondents across level sum scores on the EQ-5D-3L and EQ-5D-Y-3L (online sample, n=996)

Online resource 2 Distribution of respondents across level sum scores on the EQ-5D-3L and EQ-5D-Y-3L (CAPI sample, n=200)

Online resource 3 Distribution of respondents across index values on the EQ-5D-3L and EQ-5D-Y-3L (online sample, n = 996)

Online resource 4 Distribution of respondents across index values on the EQ-5D-3L and EQ-5D-Y-3L (CAPI sample, n = 200)

Online resource 5 Ceiling, floor, informativity and agreement (online sample, n=996)

| **Dimensions** | **Level 1 (ceiling)**  **n (%)** | | | | **Level 2**  **n (%)** | | **Level 3 (floor)**  **n (%)** | | | | **Absolute informativity (H’)** | | **Relative informativity**  **(J’)** | | **Agreement (Kendall’s tau)** |
| --- | --- | --- | --- | --- | --- | --- | --- | --- | --- | --- | --- | --- | --- | --- | --- |
|  | EQ-5D-Y-3L | EQ-5D-3L | Absolute ceiling difference (pp) | Relative ceiling difference (%) | EQ-5D-Y-3L | EQ-5D-3L | EQ-5D-Y-3L | EQ-5D-3L | Absolute floor difference (pp) | Relative floor difference (%) | EQ-5D-Y-3L | EQ-5D-3L | EQ-5D-Y-3L | EQ-5D-3L |  |
| Mobility ^a, b^ | 730 (73.3) | 748 (75.1) | 1.8 | 2.4 | 217 (21.8) | 247 (24.8) | 49 (4.9) | 1 (0.1) | -4.8 | -4800.0 | 1.02 | 0.82 | 0.64 | 0.52 | 0.835 |
| Looking after myself / Self-care | 948 (95.2) | 952 (95.6) | 0.4 | 0.4 | 38 (3.8) | 44 (4.4) | 10 (1.0) | 0 (0.0) | -1.0 | - | 0.31 | 0.26 | 0.20 | 0.16 | 0.726 |
| Doing usual activities / Usual activities ^b^ | 818 (82.1) | 835 (83.8) | 1.7 | 2.0 | 157 (15.8) | 159 (16.0) | 21 (2.1) | 2 (0.2) | -1.9 | -950.0 | 0.77 | 0.65 | 0.49 | 0.41 | 0.710 |
| Having pain/discomfort / Pain/discomfort ^a, b^ | 531 (53.3) | 589 (59.1) | 5.8 | 9.9 | 441 (44.3) | 392 (39.4) | 24 (2.4) | 15 (1.5) | -0.9 | -60.0 | 1.13 | 1.07 | 0.72 | 0.67 | 0.765 |
| Feeling worried, sad or unhappy / Anxiety/depression ^a, b^ | 533 (53.5) | 686 (68.9) | 15.4 | 22.0 | 417 (41.9) | 289 (29.0) | 46 (4.6) | 21 (2.1) | -2.5 | -119.0 | 1.21 | 1.01 | 0.77 | 0.63 | 0.627 |
| Total/average ^a^ | 311 (31.2) | 439 (44.1) | 12.9 | 29.2 | - | - | 0 (0.0) | 0 (0.0) | - | - | 0.89 | 0.76 | 0.56 | 0.48 | - |

McNemar’s test was used to assess the difference in ceiling and floor between EQ-5D-Y-3L and EQ-5D-3L. Statistically significant differences in ceiling are marked with superscript ‘a’ and differences in floor with superscript ‘b’ (p<0.05).

Online resource 6 Ceiling, floor, informativity and agreement (CAPI sample, n=200)

| **Dimensions** | **Level 1 (ceiling)**  **n (%)** | | | | **Level 2**  **n (%)** | | **Level 3 (floor)**  **n (%)** | | | | **Absolute informativity (H’)** | | **Relative informativity**  **(J’)** | | **Agreement (Kendall’s tau)** |
| --- | --- | --- | --- | --- | --- | --- | --- | --- | --- | --- | --- | --- | --- | --- | --- |
|  | EQ-5D-Y-3L | EQ-5D-3L | Absolute ceiling difference (pp) | Relative ceiling difference (%) | EQ-5D-Y-3L | EQ-5D-3L | EQ-5D-Y-3L | EQ-5D-3L | Absolute floor difference (pp) | Relative floor difference (%) | EQ-5D-Y-3L | EQ-5D-3L | EQ-5D-Y-3L | EQ-5D-3L |  |
| Mobility ^b^ | 144 (72.0) | 145 (72.5) | 0.5 | 0.7 | 40 (20.0) | 52 (26.0) | 16 (8.0) | 3 (1.5) | -6.5 | -433.3 | 1.10 | 0.93 | 0.69 | 0.59 | 0.864 |
| Looking after myself / Self-care | 191 (95.5) | 190 (95.0) | -0.5 | -0.5 | 6 (3.0) | 7 (3.5) | 3 (1.5) | 3 (1.5) | 0.0 | 0.0 | 0.31 | 0.33 | 0.19 | 0.21 | 0.840 |
| Doing usual activities / Usual activities | 173 (86.5) | 179 (89.5) | 3.0 | 3.4 | 20 (10.0) | 17 (8.5) | 7 (3.5) | 4 (2.0) | -1.5 | -75.0 | 0.68 | 0.56 | 0.43 | 0.35 | 0.780 |
| Having pain/discomfort / Pain/discomfort | 144 (72.0) | 152 (76.0) | 4.0 | 5.3 | 50 (25.0) | 45 (22.5) | 6 (3.0) | 3 (1.5) | -1.5 | -100.0 | 0.99 | 0.88 | 0.63 | 0.55 | 0.727 |
| Feeling worried, sad or unhappy / Anxiety/depression ^a^ | 146 (73.0) | 170 (85.0) | 12.0 | 14.1 | 49 (24.5) | 29 (14.5) | 5 (2.5) | 1 (0.5) | -2.0 | -400.0 | 0.96 | 0.64 | 0.61 | 0.40 | 0.654 |
| Total/average ^a^ | 105 (52.5) | 121 (60.5) | 8.0 | 13.2 | - | - | 0 (0.0) | 0 (0.0) | - | - | 0.81 | 0.67 | 0.51 | 0.42 | - |

McNemar’s test was used to assess the difference in ceiling and floor between EQ-5D-Y-3L and EQ-5D-3L. Statistically significant differences in ceiling are marked with superscript ‘a’ and differences in floor with superscript ‘b’ (p<0.05).

Online resource 7 Cross-tabulation of EQ-5D-Y-3L and EQ-5D-3L responses (online sample, n=996)

| **EQ-5D-Y-3L** | **EQ-5D-3L** | | | **Consistent response pairs (n, %)** |
| --- | --- | --- | --- | --- |
| **Mobility (Y-3L) vs. Mobility (3L), n (%)** | *I have no problems walking about* | *I have some problems walking about* | *I am confined to bed* | 891 (89.5) |
| *I have no problems walking about* | 710 (94.9%) | 20 (8.1%) | 0 (0.0%) |  |
| *I have some problems walking about* | 37 (4.9%) | 180 (72.9%) | 0 (0.0%) |  |
| *I have a lot of problems walking about* | 1 (0.1%) | 47 (19.0%) | 1 (100.0%) |  |
| **Looking after myself (Y-3L) vs. Self-care (3L), n (%)** | *I have no problems with self-care* | *I have some problems washing or dressing myself* | *I am unable to wash or dress myself* | 963 (96.7) |
| *I have no problems washing or dressing myself* | 938 (98.5%) | 10 (22.7%) | 0 (0.0%) |  |
| *I have some problems washing or dressing myself* | 13 (1.4%) | 25 (56.8%) | 0 (0.0%) |  |
| *I have a lot of problems washing or dressing myself* | 1 (0.1%) | 9 (20.5%) | 0 (0.0%) |  |
| **Doing usual activities (Y-3L) vs. Usual activities (3L), n (%)** | *I have no problems with performing my usual activities* | *I have some problems with performing my usual activities* | *I am unable to perform my usual activities* | 799 (80.2) |
| *I have no problems doing my usual activities* | 786 (94.1%) | 32 (54.2%) | 0 (0.0%) |  |
| *I have some problems doing my usual activities* | 46 (5.5%) | 11 (18.6%) | 0 (0.0%) |  |
| *I have a lot of problems doing my usual activities* | 3 (0.4%) | 16 (27.1%) | 2 (100.0%) |  |
| **Having pain/discomfort (Y-3L) vs. Pain/discomfort (3L), n (%)** | *I have no pain or discomfort* | *I have moderate pain or discomfort^1^* | *I have extreme pain or discomfort^2^* | 867 (87.1) |
| *I have no pain or discomfort* | 501 (85.1%) | 30 (7.7%) | 0 (0.0%) |  |
| *I have some pain or discomfort* | 86 (14.6%) | 353 (90.1%) | 2 (13.3%) |  |
| *I have a lot of pain or discomfort* | 2 (0.3%) | 9 (2.3%) | 13 (86.7%) |  |
| **Feeling worried, sad or unhappy (Y-3L) vs. Anxiety/depression (3L), n (%)** | *I am not anxious or depressed* | *I am moderately anxious or depressed^3^* | *I am extremely anxious or depressed^4^* | 760 (76.3) |
| *I am not worried, sad or unhappy* | 509 (74.2%) | 24 (8.3%) | 0 (0.0%) |  |
| *I am a bit worried, sad or unhappy* | 173 (25.2%) | 237 (82.0%) | 7 (33.3%) |  |
| *I am very worried, sad or unhappy* | 4 (0.6%) | 28 (9.7%) | 14 (66.7%) |  |

Percentages may not total 100 by rows due to rounding.

1-in Hungarian: I have moderate pain or a little discomfort

2-in Hungarian: I have very strong pain or very large discomfort

3-in Hungarian: I am moderately anxious or feeling down a little

4-in Hungarian: I am very much anxious or feeling down a lot

Online resource 8 Cross-tabulation of EQ-5D-Y-3L and EQ-5D-3L responses (CAPI sample, n=200)

| **EQ-5D-Y-3L** | **EQ-5D-3L** | | | **Consistent response pairs (n, %)** |
| --- | --- | --- | --- | --- |
| **Mobility (Y-3L) vs. Mobility (3L), n (%)** | *I have no problems walking about* | *I have some problems walking about* | *I am confined to bed* | 172 (86.0) |
| *I have no problems walking about* | 140 (96.6) | 4 (7.7) | 0 (0.0) |  |
| *I have some problems walking about* | 5 (3.4) | 34 (65.4) | 1 (33.3) |  |
| *I have a lot of problems walking about* | 0 (0.0) | 14 (26.9) | 2 (66.7) |  |
| **Looking after myself (Y-3L) vs. Self-care (3L), n (%)** | *I have no problems with self-care* | *I have some problems washing or dressing myself* | *I am unable to wash or dress myself* | 197 (98.5) |
| *I have no problems washing or dressing myself* | 189 (99.5) | 2 (28.6) | 0 (0.0) |  |
| *I have some problems washing or dressing myself* | 1 (0.5) | 5 (71.4) | 0 (0.0) |  |
| *I have a lot of problems washing or dressing myself* | 0 (0.0) | 0 (0.0) | 3 (100.0) |  |
| **Doing usual activities (Y-3L) vs. Usual activities (3L), n (%)** | *I have no problems with performing my usual activities* | *I have some problems with performing my usual activities* | *I am unable to perform my usual activities* | 187 (93.5) |
| *I have no problems doing my usual activities* | 171 (95.5) | 2 (11.8) | 0 (0.0) |  |
| *I have some problems doing my usual activities* | 8 (4.5) | 12 (70.6) | 0 (0.0) |  |
| *I have a lot of problems doing my usual activities* | 0 (0.0) | 3 (17.6) | 4 (100.0) |  |
| **Having pain/discomfort (Y-3L) vs. Pain/discomfort (3L), n (%)** | *I have no pain or discomfort* | *I have moderate pain or discomfort^1^* | *I have extreme pain or discomfort^2^* | 175 (87.5) |
| *I have no pain or discomfort* | 137 (90.1) | 7 (15.6) | 0 (0.0) |  |
| *I have some pain or discomfort* | 15 (9.9) | 35 (77.8) | 0 (0.0) |  |
| *I have a lot of pain or discomfort* | 0 (0.0) | 3 (6.7) | 3 (100.0) |  |
| **Feeling worried, sad or unhappy (Y-3L) vs. Anxiety/depression (3L), n (%)** | *I am not anxious or depressed* | *I am moderately anxious or depressed^3^* | *I am extremely anxious or depressed^4^* | 172 (86.0) |
| *I am not worried, sad or unhappy* | 145 (85.3) | 1 (3.4) | 0 (0.0) |  |
| *I am a bit worried, sad or unhappy* | 23 (13.5) | 26 (89.7) | 0 (0.0) |  |
| *I am very worried, sad or unhappy* | 2 (1.2) | 2 (6.9) | 1 (100.0) |  |

Percentages may not total 100 by rows due to rounding.

1-in Hungarian: I have moderate pain or a little discomfort

2-in Hungarian: I have very strong pain or very large discomfort

3-in Hungarian: I am moderately anxious or feeling down a little

4-in Hungarian: I am very much anxious or feeling down a lot

Online resource 9 Characteristics of the EQ-5D-Y-3L and EQ-5D-3L health state profiles (online sample, n=996)

|  | **EQ-5D-Y-3L** | | | **EQ-5D-3L** | | |
| --- | --- | --- | --- | --- | --- | --- |
| Theoretical number of health state profiles | 243 | | | 243 | | |
| Observed health state profiles | 76 | | | 38 | | |
| Proportion of health state profiles used (%) | 31.3 | | | 15.6 | | |
| Consistent health profiles (including ‘11111’) (n, %) | 571 (57.3%) | | | | | |
| Consistent health profiles (excluding ‘11111’) (n, %) | 279 (28.0%) | | | | | |
| Intraclass correlation coefficient for level sum scores (95% CI) | 0.799 (0.718-0.851) | | | | | |
| Intraclass correlation coefficient for index values (95% CI) | 0.720 (0.632-0.783) | | | | | |
| Floor (%) | 0 | | | 0 | | |
| Ceiling (%) | 31.2 | | | 44.1 | | |
| Shannon’s index (H’) | 3.78 | | | 3.18 | | |
| H’ max | 7.92 | | | 7.92 | | |
| Shannon’s evenness index (J’) | 0.48 | | | 0.40 | | |
| 10 most common health state profiles | **Profile** | **Frequency** | **Relative frequency (%)** | **Profile** | **Frequency** | **Relative frequency (%)** |
|  | 11111 | 311 | 31.2 | 11111 | 439 | 44.1 |
|  | 11112 | 145 | 14.6 | 11122 | 96 | 9.6 |
|  | 11122 | 136 | 13.7 | 11112 | 77 | 7.7 |
|  | 11121 | 68 | 6.8 | 11121 | 75 | 7.5 |
|  | 21121 | 43 | 4.3 | 21121 | 54 | 5.4 |
|  | 21122 | 39 | 3.9 | 21111 | 41 | 4.1 |
|  | 21111 | 31 | 3.1 | 21122 | 33 | 3.3 |
|  | 21222 | 31 | 3.1 | 21221 | 29 | 2.9 |
|  | 11222 | 15 | 1.5 | 21222 | 27 | 2.7 |
|  | 21221 | 14 | 1.4 | 11222 | 23 | 2.3 |

Intraclass correlation coefficients (ICC) were calculated using a two-way mixed-effects model with absolute agreement.

Online resource 10 Characteristics of the EQ-5D-Y-3L and EQ-5D-3L health state profiles (CAPI sample, n=200)

|  | **EQ-5D-Y-3L** | | | **EQ-5D-3L** | | |
| --- | --- | --- | --- | --- | --- | --- |
| Theoretical number of health state profiles | 243 | | | 243 | | |
| Observed health state profiles | 35 | | | 26 | | |
| Proportion of health state profiles used (%) | 14.4 | | | 10.7 | | |
| Consistent health profiles (including ‘11111’) (n, %) | 135 (67.5%) | | | | | |
| Consistent health profiles (excluding ‘11111’) (n, %) | 33 (16.5%) | | | | | |
| Intraclass correlation coefficient for level sum scores (95% CI) | 0.862 (0.788-0.906) | | | | | |
| Intraclass correlation coefficient for index values (95% CI) | 0.794 (0.697-0.856) | | | | | |
| Floor (%) | 0 | | | 0 | | |
| Ceiling (%) | 52.5 | | | 60.5 | | |
| Shannon’s index (H’) | 3.08 | | | 2.54 | | |
| H’ max | 7.92 | | | 7.92 | | |
| Shannon’s evenness index (J’) | 0.39 | | | 0.32 | | |
| 10 most common health state profiles | **Profile** | **Frequency** | **Relative frequency (%)** | **Profile** | **Frequency** | **Relative frequency (%)** |
|  | 11111 | 105 | 52.5 | 11111 | 121 | 60.5 |
|  | 11112 | 17 | 8.5 | 21121 | 14 | 7.0 |
|  | 11121 | 10 | 5.0 | 21111 | 12 | 6.0 |
|  | 21121 | 9 | 4.5 | 11122 | 8 | 4.0 |
|  | 21112 | 7 | 3.5 | 11121 | 7 | 3.5 |
|  | 21111 | 6 | 3.0 | 11112 | 5 | 2.5 |
|  | 11122 | 5 | 2.5 | 21221 | 4 | 2.0 |
|  | 21221 | 4 | 2.0 | 21112 | 4 | 2.0 |
|  | 21222 | 4 | 2.0 | 21122 | 4 | 2.0 |
|  | 21122 | 3 | 1.5 | 21211 | 3 | 1.5 |

Intraclass correlation coefficients (ICC) were calculated using a two-way mixed-effects model with absolute agreement.

Online resource 11 Bland-Altman plots comparing Y-3L and 3L index values and level sum scores (online sample, n=996)


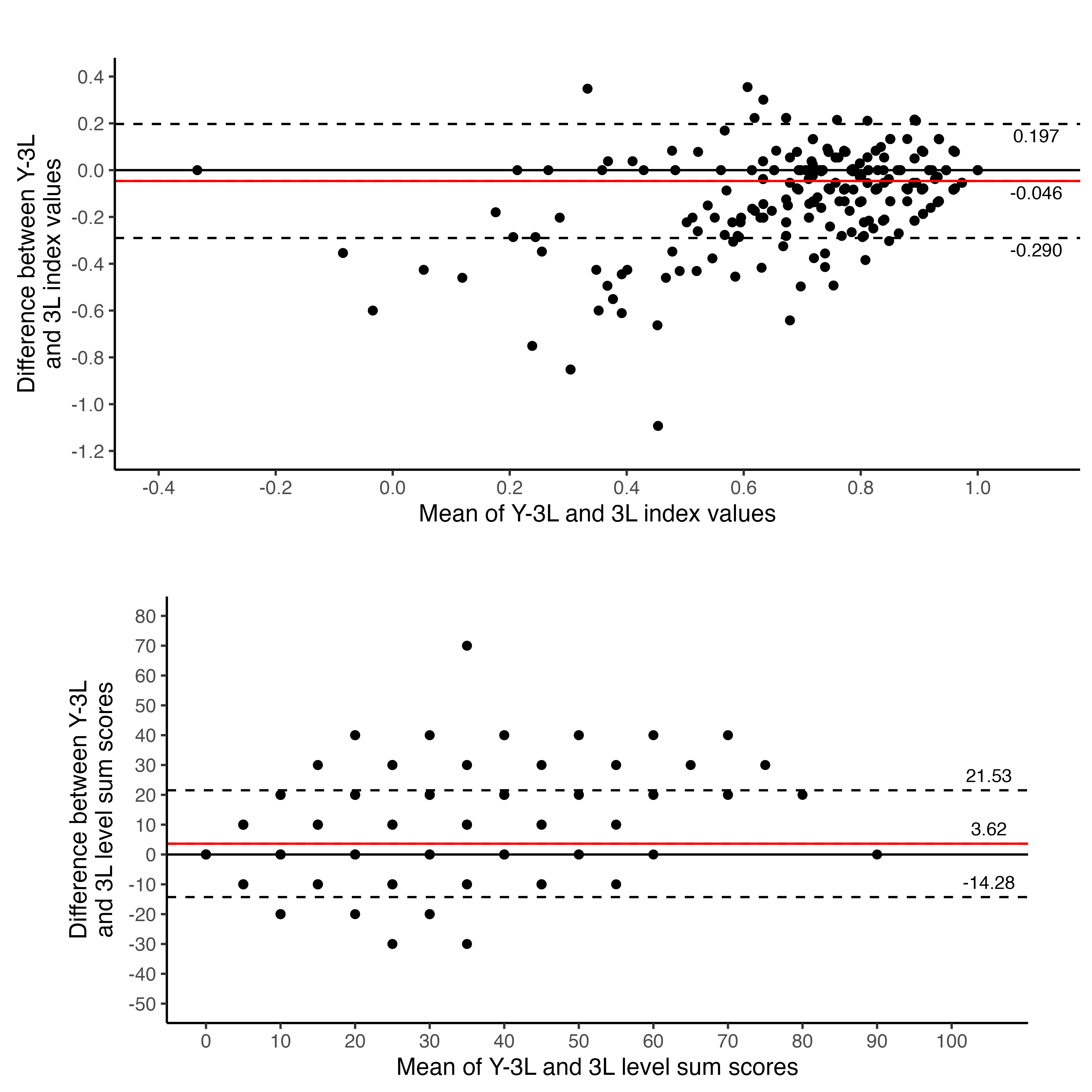


In both panels, the solid red horizontal line represents the mean difference (D) between the Y-3L and 3L index values and level sum scores. The dashed lines indicate the 95% limits of agreement, calculated as D ± 1.96 × SD, where SD is the standard deviation of the differences.

Online resource 12 Bland-Altman plots comparing Y-3L and 3L index values and level sum scores (CAPI sample, n=200)


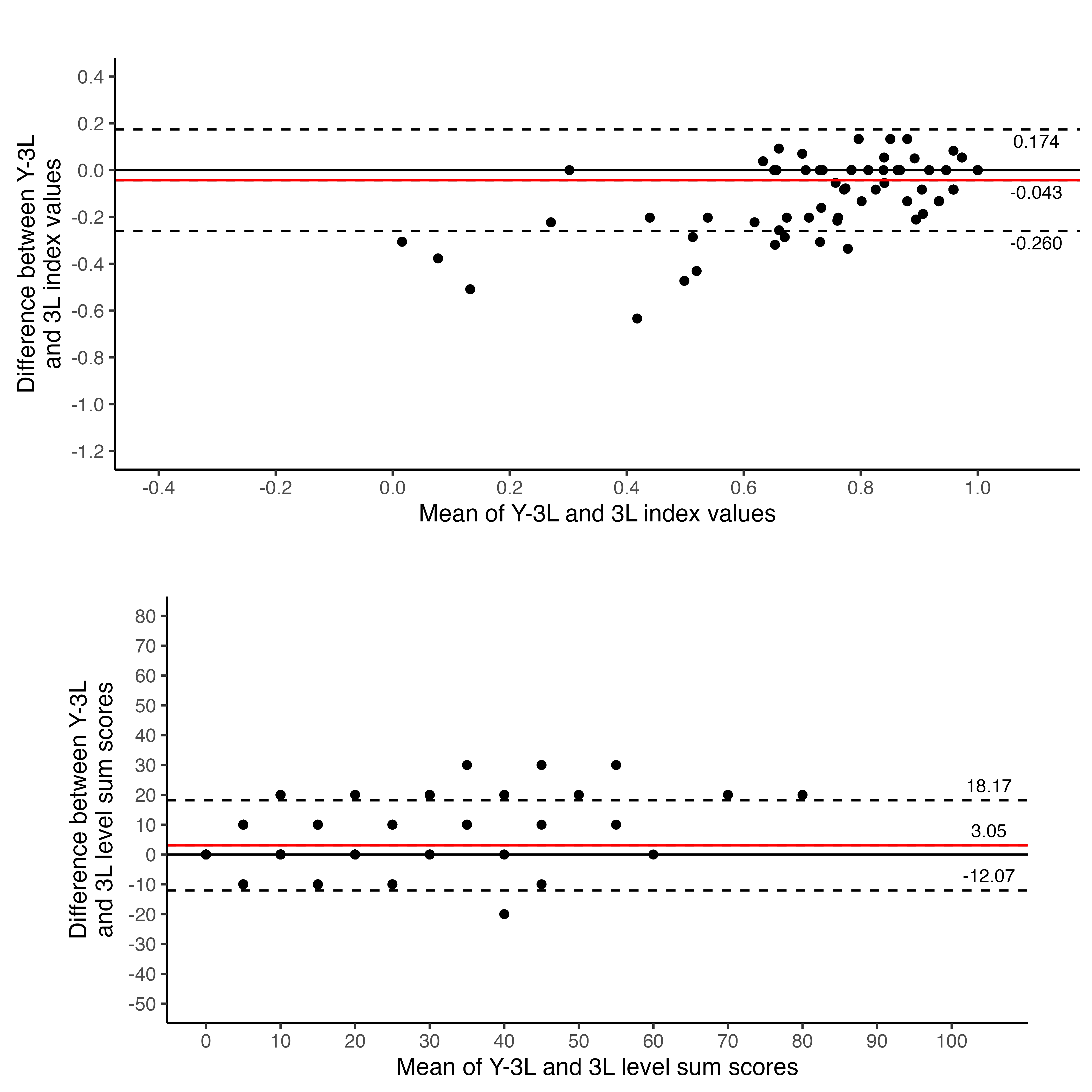


In both panels, the solid red horizontal line represents the mean difference (D) between the Y-3L and 3L index values and level sum scores. The dashed lines indicate the 95% limits of agreement, calculated as D ± 1.96 × SD, where SD is the standard deviation of the differences.

Online resource 13 Known-groups validity of level sum scores (0-100) (online sample, n=996)

|  | **n (%)** | **EQ-5D-Y-3L** | | | **EQ-5D-3L** | | | **Y-3L vs. 3L paired t-test (p-value)** | **RE ^d^** | **95% CI ^e^** |
| --- | --- | --- | --- | --- | --- | --- | --- | --- | --- | --- |
|  |  | **Mean (SD)** | **p-value** | **ES ^c^** | **Mean (SD)** | **p-value** | **ES ^c^** |  |  |  |
| **Total sample** | 996 (100.0) | 15.76 (16.34) | - | - | 12.14 (14.14) | - | - | <0.001 | - | - |
| **Self-perceived health status ^a^** |  |  |  |  |  |  |  |  |  |  |
| Excellent | 67 (6.7) | 4.48 (9.74) | <0.001 | 0.313 | 1.34 (3.85) | <0.001 | 0.315 | 0.008 | 0.994 | 0.855-1.103 |
| Very good | 252 (25.3) | 7.18 (9.59) |  |  | 4.88 (7.96) |  |  | <0.001 |  |  |
| Good | 432 (43.4) | 14.30 (12.20) |  |  | 10.80 (11.60) |  |  | <0.001 |  |  |
| Fair | 212 (21.3) | 27.00 (16.40) |  |  | 22.50 (14.60) |  |  | <0.001 |  |  |
| Poor | 33 (3.3) | 51.50 (24.00) |  |  | 40.00 (16.60) |  |  | <0.001 |  |  |
| **Health conditions ^b^** |  |  |  |  |  |  |  |  |  |  |
| Healthy | 312 (31.2) | 8.27 (9.83) | - | - | 5.58 (9.05) | - | - | <0.001 | - | - |
| Allergies | 160 (16.1) | 18.94 (17.90) | <0.001 | 0.813 | 15.31 (16.13) | <0.001 | 0.816 | <0.001 | 0.996 | 0.868-1.148 |
| Skin disease | 78 (7.8) | 19.49 (16.74) | <0.001 | 0.972 | 15.77 (15.42) | <0.001 | 0.959 | <0.001 | 1.014 | 0.866-1.222 |
| Hypertension | 305 (30.6) | 21.48 (19.60) | <0.001 | 0.854 | 16.89 (16.56) | <0.001 | 0.850 | <0.001 | 1.005 | 0.931-1.101 |
| Asthma, COPD | 56 (5.6) | 21.96 (18.33) | <0.001 | 1.189 | 19.46 (17.52) | <0.001 | 1.291 | 0.042 | 0.921 | 0.785-1.063 |
| Gastrointestinal disease | 75 (7.5) | 22.53 (18.46) | <0.001 | 1.190 | 18.67 (17.96) | <0.001 | 1.156 | 0.016 | 1.030 | 0.847-1.288 |
| Cancer | 33 (3.3) | 23.03 (14.89) | <0.001 | 1.418 | 20.61 (15.19) | <0.001 | 1.535 | 0.118 | 0.924 | 0.730-1.104 |
| Diabetes | 103 (10.3) | 24.47 (22.04) | <0.001 | 1.167 | 18.54 (18.23) | <0.001 | 1.081 | <0.001 | 1.079 | 0.960-1.244 |
| Osteoporosis | 30 (3.0) | 25.00 (21.62) | <0.001 | 1.477 | 19.67 (13.77) | <0.001 | 1.476 | 0.030 | 1.001 | 0.803-1.288 |
| Cardiovascular disease | 120 (12.1) | 26.42 (23.15) | <0.001 | 1.229 | 21.00 (18.76) | <0.001 | 1.232 | <0.001 | 0.997 | 0.905-1.105 |
| Musculoskeletal diseases | 239 (24.0) | 27.95 (19.22) | <0.001 | 1.343 | 22.68 (15.84) | <0.001 | 1.373 | <0.001 | 0.978 | 0.907-1.062 |
| ***Anxiety*** | ***78 (7.8)*** | ***31.15 (21.74)*** | ***<0.001*** | ***1.749*** | ***28.72 (16.78)*** | ***<0.001*** | ***2.099*** | ***0.100*** | ***0.833*** | ***0.741-0.921*** |
| Depression | 53 (5.3) | 36.04 (22.30) | <0.001 | 2.237 | 32.075 (16.91) | <0.001 | 2.513 | 0.037 | 0.890 | 0.776-1.001 |

COPD chronic obstructive pulmonary disease, ES effect size, RE relative efficiency.

Bold italic rows indicate statistically significant differences in relative efficiency (RE), where one instrument performed significantly better (95% CI does not include 1).

^a^ Analysis of variance to test the difference between categories, where p < 0.05 was considered statistically significant.

^b^ Student’s t-test compared to the healthy subgroup, where p < 0.05 was considered statistically significant.

^c^ Effect sizes were calculated using Cohen’s d (two groups) or η^2^ (three or more groups).

^d^ Relative efficiency compared to EQ-5D-3L.

^e^ 2000 bootstrap samples with accelerated bias correction.

Online resource 14 Known-groups validity of level sum scores (0-100) (CAPI sample, n=200)

|  | **n (%)** | **EQ-5D-Y-3L** | | | **EQ-5D-3L** | | | **Y-3L vs. 3L paired t-test (p-value)** | **RE ^d^** | **95% CI ^e^** |
| --- | --- | --- | --- | --- | --- | --- | --- | --- | --- | --- |
|  |  | **Mean (SD)** | **p-value** | **ES ^c^** | **Mean (SD)** | **p-value** | **ES ^c^** |  |  |  |
| **Total sample** | 200 (100.0) | 11.95 (17.21) | - | - | 8.90 (13.78) | - | - | 0.051 | - | - |
| **Self-perceived health status ^a^** |  |  |  |  |  |  |  |  |  |  |
| Excellent | 35 (17.5) | 2.00 (4.73) | <0.001 | 0.395 | 0.86 (2.84) | <0.001 | 0.388 | 0.044 | 1.018 | 0.871-1.182 |
| Very good | 69 (34.5) | 4.78 (7.79) |  |  | 2.90 (6.44) |  |  | <0.001 |  |  |
| Good | 57 (28.5) | 12.10 (14.10) |  |  | 9.30 (12.80) |  |  | 0.005 |  |  |
| Fair | 32 (16.0) | 29.70 (20.70) |  |  | 24.70 (15.20) |  |  | 0.033 |  |  |
| Poor | 7 (3.5) | 50.00 (20.00) |  |  | 32.90 (16.00) |  |  | 0.003 |  |  |
| **Health conditions ^b^** |  |  |  |  |  |  |  |  |  |  |
| Healthy | 88 (44.0) | 4.20 (7.54) | - | - | 2.96 (6.64) | - | - | 0.245 | - | - |
| Allergies | 20 (10.0) | 8.00 (12.40) | <0.001 | 0.441 | 6.50 (10.89) | <0.001 | 0.468 | 0.687 | 0.942 | -0.416-11.494 |
| Skin disease | 10 (5.0) | 7.00 (13.38) | <0.001 | 0.338 | 5.00 (8.50) | <0.001 | 0.299 | 0.695 | 1.130 | -4.309-5.214 |
| Hypertension | 55 (27.5) | 20.73 (20.54) | <0.001 | 1.179 | 16.00 (16.17) | <0.001 | 1.156 | 0.183 | 1.019 | 0.850-1.279 |
| Asthma, COPD | 9 (4.5) | 15.56 (20.07) | <0.001 | 1.224 | 11.11 (13.64) | <0.001 | 1.090 | 0.591 | 1.123 | NA |
| Gastrointestinal disease | 13 (6.5) | 20.77 (20.60) | <0.001 | 1.645 | 16.15 (12.61) | <0.001 | 1.734 | 0.499 | 0.949 | 0.619-1.363 |
| Cancer | 8 (4.0) | 33.75 (5.18) | <0.001 | 3.999 | 25.00 (9.26) | <0.001 | 3.211 | 0.040 | 1.246 | NA |
| Diabetes | 15 (7.5) | 30.00 (25.07) | <0.001 | 2.211 | 24.67 (18.07) | <0.001 | 2.380 | 0.510 | 0.929 | 0.707-1.181 |
| Osteoporosis | 7 (3.5) | 28.57 (22.68) | <0.001 | 2.622 | 21.43 (15.74) | <0.001 | 2.443 | 0.508 | 1.073 | NA |
| Cardiovascular disease | 19 (9.5) | 42.11 (22.75) | <0.001 | 3.252 | 29.47 (17.47) | <0.001 | 2.814 | 0.063 | 1.156 | 0.905-1.492 |
| Musculoskeletal diseases | 40 (20.0) | 31.75 (19.73) | <0.001 | 2.180 | 23.00 (15.56) | <0.001 | 1.953 | 0.031 | 1.116 | 0.941-1.351 |
| Anxiety | 3 (1.5) | 36.67 (5.77) | <0.001 | 4.326 | 36.67 (11.55) | <0.001 | 4.968 | 1.000 | 0.871 | NA |
| Depression | 3 (1.5) | 36.67 (5.77) | <0.001 | 0.436 | 26.67 (5.77) | <0.001 | 3.583 | 0.101 | 1.208 | NA |

COPD chronic obstructive pulmonary disease, ES effect size, NA not applicable, RE relative efficiency.

^a^ Analysis of variance to test the difference between categories, where p < 0.05 was considered statistically significant.

^b^ Student’s t-test compared to the healthy subgroup, where p < 0.05 was considered statistically significant.

^c^ Effect sizes were calculated using Cohen’s d (two groups) or η^2^ (three or more groups).

^d^ Relative efficiency compared to EQ-5D-3L.

^e^ 2000 bootstrap samples with accelerated bias correction.

Online resource 15 Known-groups validity of index values (online sample, n=996)

|  | **n (%)** | **EQ-5D-Y-3L** | | | **EQ-5D-3L** | | | **Y-3L vs. 3L paired t-test (p-value)** | **RE ^d^** | **95% CI ^e^** |
| --- | --- | --- | --- | --- | --- | --- | --- | --- | --- | --- |
|  |  | **Mean (SD)** | **p-value** | **ES ^c^** | **Mean (SD)** | **p-value** | **ES ^c^** |  |  |  |
| **Total sample** | 996 (100.0) | 0.835 (0.197) | - | - | 0.882 (0.148) | - | - | <0.001 | - | - |
| **Self-perceived health status ^a^** |  |  |  |  |  |  |  |  |  |  |
| Excellent | 67 (6.7) | 0.952 (0.139) | <0.001 | 0.284 | 0.986 (0.041) | <0.001 | 0.298 | 0.049 | 0.954 | 0.740-1.077 |
| Very good | 252 (25.3) | 0.927 (0.102) |  |  | 0.953 (0.079) |  |  | <0.001 |  |  |
| Good | 432 (43.4) | 0.860 (0.127) |  |  | 0.897 (0.110) |  |  | <0.001 |  |  |
| Fair | 212 (21.3) | 0.715 (0.207) |  |  | 0.782 (0.156) |  |  | <0.001 |  |  |
| Poor | 33 (3.3) | 0.344 (0.375) |  |  | 0.564 (0.255) |  |  | <0.001 |  |  |
| **Health conditions ^b^** |  |  |  |  |  |  |  |  |  |  |
| Healthy | 312 (31.2) | 0.916 (0.108) | - | - | 0.943 (0.099) | - | - | 0.001 | - | - |
| Allergies | 160 (16.1) | 0.805 (0.210) | <0.001 | 0.734 | 0.851 (0.168) | <0.001 | 0.729 | <0.001 | 1.007 | 0.879-1.167 |
| Skin disease | 78 (7.8) | 0.799 (0.191) | <0.001 | 0.910 | 0.848 (0.143) | <0.001 | 0.873 | 0.002 | 1.042 | 0.872-1.326 |
| Hypertension | 305 (30.6) | 0.722 (0.249) | <0.001 | 0.749 | 0.835 (0.181) | <0.001 | 0.741 | <0.001 | 1.011 | 0.933-1.126 |
| Asthma, COPD | 56 (5.6) | 0.765 (0.239) | <0.001 | 1.108 | 0.800 (0.218) | <0.001 | 1.154 | 0.055 | 0.961 | 0.777-1.157 |
| Gastrointestinal disease | 75 (7.5) | 0.760 (0.230) | <0.001 | 1.114 | 0.810 (0.207) | <0.001 | 1.046 | 0.019 | 1.066 | 0.871-1.359 |
| Cancer | 33 (3.3) | 0.778 (0.167) | <0.001 | 1.204 | 0.804 (0.169) | <0.001 | 1.302 | 0.133 | 0.925 | 0.722-1.163 |
| ***Diabetes*** | ***103 (10.3)*** | ***0.730 (0.293)*** | ***<0.001*** | ***1.075*** | ***0.820 (0.203)*** | ***<0.001*** | ***0.931*** | ***<0.001*** | ***1.154*** | ***1.014-1.362*** |
| Osteoporosis | 30 (3.0) | 0.745 (0.259) | <0.001 | 1.331 | 0.815 (0.128) | <0.001 | 1.261 | 0.081 | 1.056 | 0.773-1.575 |
| Cardiovascular disease | 120 (12.1) | 0.700 (0.305) | <0.001 | 1.166 | 0.791 (0.210) | <0.001 | 1.096 | <0.001 | 1.064 | 0.952-1.193 |
| Musculoskeletal diseases | 239 (24.0) | 0.705 (0.254) | <0.001 | 1.135 | 0.785 (0.174) | <0.001 | 1.154 | <0.001 | 0.983 | 0.901-1.083 |
| Anxiety | 78 (7.8) | 0.647 (0.298) | <0.001 | 1.636 | 0.712 (0.206) | <0.001 | 1.815 | 0.009 | 0.901 | 0.784-1.044 |
| Depression | 53 (5.3) | 0.597 (0.300) | <0.001 | 2.107 | 0.679 (0.215) | <0.001 | 2.156 | 0.008 | 0.977 | 0.834-1.151 |

COPD chronic obstructive pulmonary disease, ES effect size, RE relative efficiency.

Bold italic rows indicate statistically significant differences in relative efficiency (RE), where one instrument performed significantly better (95% CI does not include 1).

^a^ Analysis of variance to test the difference between categories, where p < 0.05 was considered statistically significant.

^b^ Student’s t-test compared to the healthy subgroup, where p < 0.05 was considered statistically significant.

^c^ Effect sizes were calculated using Cohen’s d (two groups) or η^2^ (three or more groups).

^d^ Relative efficiency compared to EQ-5D-3L.

^e^ 2000 bootstrap samples with accelerated bias correction.

Online resource 16 Known-groups validity of index values (CAPI sample, n=200)

|  | **n (%)** | **EQ-5D-Y-3L** | | | **EQ-5D-3L** | | | **Y-3L vs. 3L paired t-test (p-value)** | **RE ^d^** | **95% CI ^e^** |
| --- | --- | --- | --- | --- | --- | --- | --- | --- | --- | --- |
|  |  | **Mean (SD)** | **p-value** | **ES ^c^** | **Mean (SD)** | **p-value** | **ES ^c^** |  |  |  |
| **Total sample** | 200 (100.0) | 0.872 (0.214) | - | - | 0.916 (0.144) | - | - | 0.019 | - | - |
| **Self-perceived health status ^a^** |  |  |  |  |  |  |  |  |  |  |
| Excellent | 35 (17.5) | 0.981 (0.047) | <0.001 | 0.364 | 0.993 (0.025) | <0.001 | 0.351 | 0.050 | 1.036 | 0.866-1.275 |
| Very good | 69 (34.5) | 0.958 (0.071) |  |  | 0.975 (0.060) |  |  | 0.002 |  |  |
| Good | 57 (28.5) | 0.884 (0.140) |  |  | 0.915 (0.112) |  |  | 0.003 |  |  |
| Fair | 32 (16.0) | 0.661 (0.296) |  |  | 0.761 (0.188) |  |  | 0.007 |  |  |
| Poor | 7 (3.5) | 0.362 (0.343) |  |  | 0.654 (0.248) |  |  | 0.003 |  |  |
| **Health conditions ^b^** |  |  |  |  |  |  |  |  |  |  |
| Healthy | 88 (44.0) | 0.963 (0.074) | - | - | 0.974 (0.062) | - | - | 0.279 | - | - |
| Allergies | 20 (10.0) | 0.916 (0.158) | <0.001 | 0.494 | 0.939 (0.100) | <0.001 | 0.499 | 0.387 | 0.991 | -0.525-7.966 |
| Skin disease | 10 (5.0) | 0.918 (0.155) | <0.001 | 0.520 | 0.944 (0.093) | <0.001 | 0.452 | 0.343 | 1.149 | -4.198-5.427 |
| Hypertension | 55 (27.5) | 0.864 (0.285) | <0.001 | 1.068 | 0.843 (0.184) | <0.001 | 1.053 | <0.001 | 1.014 | 0.836-1.245 |
| Asthma, COPD | 9 (4.5) | 0.839 (0.241) | <0.001 | 1.244 | 0.905 (0.120) | <0.001 | 0.998 | 0.262 | 1.247 | -0.137-4.488 |
| Gastrointestinal disease | 13 (6.5) | 0.728 (0.312) | <0.001 | 1.819 | 0.823 (0.165) | <0.001 | 1.844 | 0.112 | 0.987 | 0.638-1.424 |
| Cancer | 8 (4.0) | 0.658 (0.080) | <0.001 | 4.086 | 0.785 (0.098) | <0.001 | 2.891 | 0.054 | 1.413 | NA |
| Diabetes | 15 (7.5) | 0.627 (0.378) | <0.001 | 2.138 | 0.746 (0.209) | <0.001 | 2.355 | 0.065 | 0.908 | 0.670-1.211 |
| Osteoporosis | 7 (3.5) | 0.623 (0.364) | <0.001 | 2.906 | 0.764 (0.216) | <0.001 | 2.572 | 0.176 | 1.130 | NA |
| Cardiovascular disease | 19 (9.5) | 0.474 (0.350) | <0.001 | 3.057 | 0.691 (0.233) | <0.001 | 2.527 | <0.001 | 1.210 | 0.922-1.573 |
| Musculoskeletal diseases | 40 (20.0) | 0.633 (0.294) | <0.001 | 1.882 | 0.776 (0.194) | <0.001 | 1.651 | <0.001 | 1.140 | 0.951-1.400 |
| Anxiety | 3 (1.5) | 0.606 (0.152) | <0.001 | 4.650 | 0.661 (0.061) | <0.001 | 5.042 | 0.539 | 0.922 | NA |
| Depression | 3 (1.5) | 0.495 (0.266) | <0.001 | 5.615 | 0.720 (0.073) | <0.001 | 4.081 | 0.243 | 1.376 | NA |

COPD chronic obstructive pulmonary disease, ES effect size, NA not applicable, RE relative efficiency.

^a^ Analysis of variance to test the difference between categories, where p < 0.05 was considered statistically significant.

^b^ Student’s t-test compared to the healthy subgroup, where p < 0.05 was considered statistically significant.

^c^ Effect sizes were calculated using Cohen’s d (two groups) or η^2^ (three or more groups).

^d^ Relative efficiency compared to EQ-5D-3L.

^e^ 2000 bootstrap samples with accelerated bias correction.
